# Shared decision-making and behaviour change collide: an analysis of consultations discussing clinical trial recruitment

**DOI:** 10.1186/s13063-025-09260-4

**Published:** 2025-11-24

**Authors:** Abigail Stephen, Ayodeji Matuluko, Kelly Carroll, Taylor Coffey, Louisa Lawrie, Frances Sherratt, Natasha Hudek, Fabianna Lorencatto, Justin Presseau, Susan Marlin, Dawn P. Richards, Jamie Brehaut, Katie Gillies

**Affiliations:** 1 Aberdeen Centre for Evaluation, University of Aberdeen, Aberdeen, UK; 2 Methodological and Implementation Research, Ottawa Hospital Research Institute, Clinical Epidemiology Program, Ottawa, ON Canada; 3 Department of Public Health, Policy & Systems, University of Liverpool, Liverpool, UK; 4 Centre for Behaviour Change, University College London, London, UK; 5 School of Epidemiology & Public Health, University of Ottawa, Ottawa, Canada; 6 Clinical Trials Ontario, Toronto, Canada

**Keywords:** Behavioural science, Clinical trials, Recruitment, Shared decision-making, Behaviour change, Informed consent

## Abstract

**Background:**

Recruitment to clinical trials is often challenging, and evidence on effective recruitment strategies remains limited. Framing trial recruitment as a behaviour, amenable to change, enables the application of behavioural science frameworks to better understand and potentially improve recruitment processes. Since trial participation is also a preference-sensitive decision, shared decision-making (SDM) may further enhance ethical recruitment. The present study aimed to explore how behavioural taxonomies and SDM frameworks can be applied to recruitment consultations to identify behaviour change techniques (BCTs) and assess the extent of SDM.

**Methods:**

A secondary analysis was conducted on 53 audio-recorded consultations; consultations from 3 trials in oncology and gastroenterology were sampled. The action, actor, context, target, and time (AACTT) framework was used to define recruitment behaviours, which were then coded using the Behaviour Change Technique Taxonomy v1 (BCTTv1). SDM was assessed using the 5-item ‘Observing Patient Involvement’ (OPTION5) tool, which evaluates patient involvement in decision-making. Descriptive statistics and frequency counts were used to analyse the data.

**Results:**

Twenty-one of the 93 BCTs in the BCTTv1 were identified across all consultations. The most frequently coded BCTs were 5.1 (information about health consequences) and 5.3 (information about social and environmental consequences), both present in all consultations. No substantial difference in the total number of BCTs was observed between trial consenters (*M* = 6.8, *SD* = 1.5) and decliners (*M* = 6.4, *SD* = 2.1). However, some techniques showed variability: BCT 7.1 (prompts and cues) appeared more frequently in consultations with consenters (84%) than decliners (38%), while BCT 1.4 (action planning) was more frequent in decliners (44%) than consenters (27%). SDM, as measured by OPTION5, was low overall, with a mean score of 27.7 (*SD* = 12.2) out of 100, with no significant differences across trials or participant groups.

**Conclusions:**

This study demonstrates the feasibility of applying behavioural science and SDM frameworks to analyse trial recruitment consultations. While a range of BCTs were identified, SDM efforts were generally low. Recruitment practices may benefit from more deliberate consideration of these techniques and greater emphasis on shared decision-making to support informed choices.

**Supplementary Information:**

The online version contains supplementary material available at 10.1186/s13063-025-09260-4.

## Background

Recruitment to clinical trials is often difficult. Successful recruitment of participants is key to ensuring trials are scientifically robust and financially viable [1, 2]. However, as many as 70% of trials funded by the National Institute for Health and Care Research in the UK fail to achieve their initial recruitment targets [1]. It is estimated that annually, less than a third of clinical trials recruit their original target sample size within the specified timeframe, and an additional third of trials require time extensions because of failures to meet recruitment goals [3]. There is substantial existing research that aims to understand, and improve on, factors affecting recruitment from both participant and trial staff perspectives [4]. Despite significant methodological efforts to understand and enhance recruitment, evidence on effective recruitment practices remains limited [5, 6].

Much of the previous research has not conceptualised these processes through a behavioural lens. During the recruitment stage of a clinical trial, potential participants attend routine consultations where a clinical trial is discussed with healthcare professionals, who perform many behaviours, such as approaching eligible participants, describing randomisation, or obtaining informed consent. By reframing trial recruitment processes as behaviours, we could obtain a better understanding of the challenges to recruitment by applying taxonomies and frameworks from behavioural science. These can provide a structured approach to understanding how individuals make decisions about trial participation and could provide direction to develop more effective strategies for recruitment [7].

The ethical principles of informed choice are key when considering participation in a clinical trial, and using a behavioural lens may help better understand decisions about trial participation [8]. Existing behavioural research has demonstrated that making informed choices for preference-sensitive decisions (i.e. a decision for which there is equivocal evidence and patient preferences should be explored) can benefit from shared decision-making (SDM) [9]. Trial participation decisions can also be considered as preference-sensitive and therefore could also benefit from SDM. Recruitment consultations in which trial participation is discussed can therefore be understood through two lenses: behaviour change, which frames trial participation as a behaviour amenable to change, and SDM, which seeks to improve the process of making difficult health decisions by emphasising deliberative, supported decision-making [10].

Recorded consultations that discuss clinical trial participation have historically been analysed using traditional qualitative approaches [11]. Given that consultations are likely to involve a range of behaviours from various people, it is of interest to explore whether behaviour change techniques (BCTs) are employed within them and whether BCTs have an effect on recruitment. BCTs are considered ‘active ingredients’ in behaviour change interventions, and the taxonomy aims to describe the elements of behaviour change. However, to also ensure principles of informed choice are being supported, considering how these consultations meet (or do not meet) standards identified for SDM that support informed choice is also important. Whereas BCTs may help to identify factors that guide recruitment consultation outcomes, assessing levels of SDM will help us to understand if decisions can benefit from support. Using these two lenses can help identify complementary approaches to support decisions about trial participation, both in terms of the behavioural factors and shared decision-making. By identifying which BCTs are implicitly used during consultations and assessing how well the conversations align with SDM principles, we may gain a better understanding of how recruitment might be improved.

This study aimed to generate understanding of trial participation by analysing audio-recorded consultations in which participation in a clinical trial is discussed (henceforth referred to as consultations), through two novel lenses: behavioural science and SDM frameworks. Specifically, it sought to answer ‘How can behavioural and shared decision-making frameworks help us understand communication and decision outcomes during consultations about clinical trial participation?’.

## Methods

### Design and data collection

We conducted a secondary analysis on a sample of anonymised transcripts from consultations where discussions around participation in clinical trials took place between healthcare professionals and potential participants. Healthcare professionals included consultants and research nurses. Transcripts were sampled from three different clinical trials (described in Tables 1 and 2), using purposive and convenience sampling strategies. The included trials were set within oncology and gastroenterology and were testing interventions including surgery, immunotherapy, and radiotherapy. Participants in the original trials consented to an audio-recorded consultation and consented for their data to be used in future secondary analyses. Only healthcare professional recruitment behaviours were coded and analysed. To ensure maximum representation within the total sample, a researcher selected trials to ensure different clinical contexts, interventions, and frequency of consent to trial decisions, meaning those who consented or declined participation in the main clinical trial following the consultation were captured in the sample. In the present study, the term ‘consenters’ is used to describe those that consented to participate in the main clinical trial following the consultation. The term ‘decliners’ is used to describe those that did not consent to participate in the trial.

**Table 1:** Characteristics of the included trials

Trial	PICO	Recruitment rate	Number of consultations	Consenters vs decliners	Gender and age of potential participants		
A	*Population*: Adults with uncomplicated symptomatic gallstone disease *Intervention and comparator*: Surgery versus no surgery *Outcome*: Pain	32%	Twenty-one from 3 sites	Consenters only		**Consenter**	**Decliner**
					**Age** **Median (range)**	49 (26–74)	-
					**Gender**	65% women	-
B	*Population*: PET-guided, response-adapted therapy in patients with previously untreated, high tumour burden follicular lymphoma *Intervention and comparator*: Rituximab maintenance with or without lenalidomide *Outcome*: Progression-free survival	Trial ongoing	Twelve from 5 sites	Six consenters Six decliners		**Consenter**	**Decliner**
					**Age** **Median (range)**	54 (44–72)	55.5 (40–73)
					**Gender**	33% women	33% women
C	*Population*: Patients who have undergone gross total surgical resection of atypical (grade II) meningioma *Intervention and comparator*: Early adjuvant fractionated radiotherapy for 6 weeks or active monitoring *Outcome:* Recurrence	25%	Twenty from 3 sites	Ten consenters Ten decliners		**Consenter**	**Decliner**
					**Age** **Median (range)**	48.5 (45–72)	57 (29–78)
					**Gender**	70% women	70% women

**Table 2:** AACTT Specified Behaviours

**Action**	**Actor**	**Context**	**Target **	**Time**
1) Discussing the option of taking part in the trial or not 2) Describing the trial interventions or alternatives (e.g., standard care), which may include describing trial rationale 3) Discussing the trial during the trial recruitment period(Could be the initial consultation or follow up ones) and/or the interventions in a balanced way 4) Exploring preferences (e.g., if the patient expresses a preference for an intervention or standard care, the trial staff probes into this/asks questions) 5) Providing information about what involvement in the trial entails and trial related activities (e.g. questionnaires, visits, how to take the intervention) 6) Seeking consent from patients (e.g. consider voluntariness (i.e. not obliged) and autonomy (i.e. up to you to make decision)) 7) Discussing or enacting (potential) randomisation 8) Exploring and/or checking patients’ acceptance of allocation (before or after randomisation) and includes potential to cross-over or withdraw	*Trial staff* (Healthcare professionals or research/other trial staff e.g., research coordinator)	*Hospitals, clinics*	*Patients eligible for trial participation*, i.e., randomisation	*During the trial recruitment period* (Could be the initial consultation or follow up ones)

### Data analysis

Only healthcare professional recruitment behaviours (as opposed to patient behaviours) were coded and analysed in the BCT coding. The present study utilises three different frameworks.

- Action, actor, context, target, and timing (AACTT) framework — A behavioural specification tool that breaks down behaviours into five components clarifying who did what, when, where, and for whom
- Behaviour Change Technique (BCT) taxonomy — A standardised and hierarchically structured list of 93 techniques that can be used to change and understand behaviour
- Observing Patient Involvement (OPTION5) tool — A 5-item tool for measuring a patient’s involvement in clinical/healthcare orientated decision-making

### Behavioural specification

The AACTT (action, actor, context, target, and timing) framework [12] was used to specify healthcare professional behaviours within the recruitment phase. The AACTT framework allowed for consistent description of discrete behaviours relevant for recruitment across each trial. Two researchers (A. M. and K. G.) used the AACTT framework to specify potential recruitment behaviours performed by healthcare professionals during a single sample consultation where a trial is discussed. This exercise ensured BCT coding was specific to recruitment behaviours by removing irrelevant, non-trial-related parts of the transcript. Each transcript was then subsequently AACTT-specified by A. M. in Microsoft Word by highlighting the relevant parts of the transcript, which were then transferred to a Microsoft Excel file for BCT coding. K. G. checked a selected 20% of the AACTT-specified transcripts to ensure consistency. Only these highlighted parts of the transcript were then BCT coded.

### Behaviour change technique coding

The Behaviour Change Technique Taxonomy v1 (BCTTv1) was used to identify the specific techniques healthcare professionals used to support recruitment efforts [13]. The AACTT-specified data excerpts from each transcript were coded to BCTs by three researchers (A. S., T. C., A. M.) in Microsoft Excel. All three coders were experienced in using this taxonomy and had previously completed the online BCTTv1 training course [14]. A pilot coding exercise was conducted by A. M. and T. C., each coding a single transcript from each of the three trials. This coding was conducted using a generic coding manual to guide decision rules. Through piloting and discussion, a project-specific coding manual was developed and iteratively updated to aid and support coding. Transcripts from all three trials were double-coded by two of the three trained BCT coders, and any conflicts or discrepancies in coding were discussed with at least one additional researcher until agreement was reached. Trials A and B were coded by A. S. and T. C., and discrepancies were discussed with K. G. Trial C was coded by A. M. and A. S., and discrepancies were discussed with T. C. and K. G. N. H. and J. P. assisted with coding queries that could not be immediately resolved through discussion with T. C. and K. G.

Data relating to BCT coding was analysed using descriptive statistics (frequency counts, means, and standard deviations) across data sets. A separate correlation analysis was conducted to assess whether the length of consultation was associated with the number of BCTs present. A word count for each consultation was calculated as a proxy for the length of consultation. The word count was calculated from the parts of the transcript highlighted during behavioural specification to ensure that only healthcare professional behaviours were captured and that consultations were comparable.

### OPTION5 rating of shared decision-making

The OPTION5 (Observing Patient Involvement) framework was used to assess SDM within the consultations. This measure comprises five items: (1) conveying the existence of options and expressing the need for a decision about trial participation, (2) supporting the patient in becoming informed about the options, (3) presenting risks and benefits of each option and ensuring understanding, (4) eliciting and understanding patient preferences, and (5) integrating preferences about trial participation decisions. Items are scored from 0 (no effort) to 4 (exemplary effort), giving a total maximum score of 20. Each transcript was assigned a score from 0 to 20 and rescaled to 0–100 following the guidance in the OPTION5 manual. OPTION5 coding of each transcript was conducted by two coders (L. L. and K. C. in the first phase and then K. G. and K. C. in the second phase). L. L. and K. C. pilot tested the coding manual on six transcripts from one trial to ensure consistency in coding and to refine the manual. Both coders independently rated a sample of 20 transcripts using the OPTION5 coding manual. Coding was compared and discussed for consensus, and a coding rulebook (available on request) was developed and updated iteratively to ensure consistency between coders. This rulebook incorporated a scoring guide and descriptors of the five items within the measure. The remaining transcripts were double-coded using the rulebook, with discussions to resolve any conflicts in coding. Coding was completed in Microsoft Word, highlighting the areas of the transcript relevant to each OPTION5 item; this coding was subsequently recorded in Microsoft Excel. Means (M) and standard deviations (SD) for each trial were calculated for all participants and for consenters and decliners sub-groups.

## Results

A total of 53 anonymised consultations where clinical trial recruitment was discussed were included. Potential trial participants included in the consultations analysed included both women and men, ranging in age from 26 to 78 years, and trial consenters and decliners. The characteristics of these trials and the participants included in the consultations analysed are described in Table 1. In the entire sample, there were 37 consenters and 16 decliners.

### AACTT specification and allocation to specified behaviours

Eight behaviours were identified and determined as relevant. Each transcript was then coded against these eight behaviours (Table 2). The actor, context, target, and timing were the same for all eight behaviours, and only the actions differed. The recruitment action of ‘describing the trial interventions or alternatives’ was the most frequently coded action (100% of consultations, *n* = 53), whereas the action of ‘exploring preferences’ was coded the least (17% of consultations, *n* = 17). A summary of all action coding across the consultations is presented in Table 3.

**Table 3:** Summary of allocation of transcripts to ‘action’ behaviours

**Action**	**Number of consultations coded to action**			**Total (%)** **(N=53)**
	**Trial**			
	**A (%) (n=21)**	**B (%) (n=20)**	**C (%) (n=12)**	
**Discussing the option of taking part in the trial or not**	17 (81%)	19 (95%)	10 (83%)	**46 (87%)**
**Describing the trial interventions or alternatives (e.g., standard care), which may include describing trial rationale**	21 (100%)	20 (100%)	12 (100%)	**53 (100%)**
**Discussing the trial and/or the interventions in a balanced way**	18 (86%)	20 (100%)	11 (92%)	**49 (92%)**
**Exploring preferences (e.g., if the patient expresses a preference for an intervention or standard care, the trial staff probes into this/asks questions)**	8 (38%)	8 (40%)	1 (8%)	**17 (32%)**
**Providing information about what involvement in the trial entails and trial related activities (e.g. questionnaires, visits, how to take the intervention)**	18 (86%)	19 (95%)	9 (75%)	**46 (87%)**
**Seeking consent from patients (e.g. consider voluntariness (i.e. not obliged) and autonomy (i.e. up to you to make decision))**	21 (100%)	20 (100%)	10 (83%)	**51 (96%)**
**Discussing or enacting (potential) randomisation**	21 (100%)	19 (95%)	5 (42%)	**45 (85%)**
**Exploring and/or checking patients’ acceptance of allocation (before or after randomisation) and includes potential to cross-over or withdraw**	20 (95%)	7 (35%)	3 (25%)	**30 (57%)**

### Presence of BCTs within consultations

Twenty-one of the 93 BCTs in the BCT Taxonomy v1 were identified across the consultations. A coding guide with examples of the BCT coding and decision rules is available in Appendix A.

### Frequency of BCTs

**Fig. 1:**
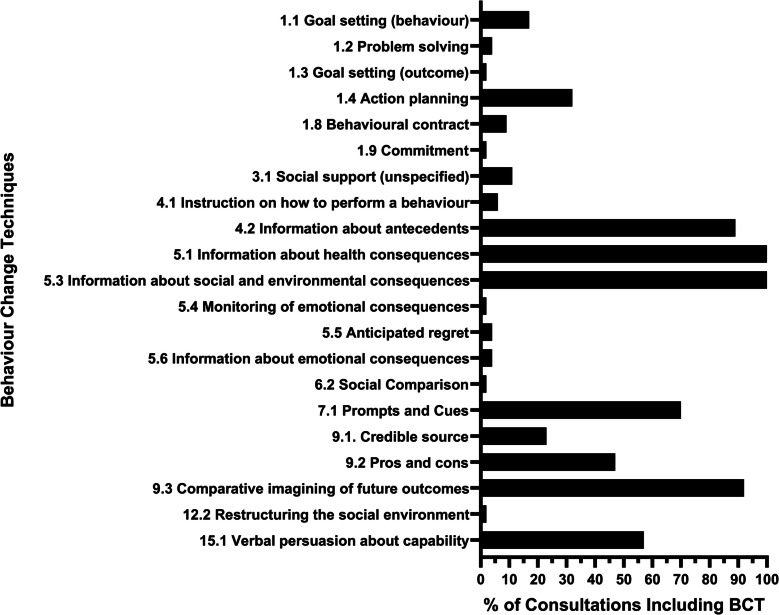
Frequency of BCTs

The most commonly identified BCTs across all consultations were 5.1 (information about health consequences) and 5.3 (information about social and environmental consequences), which were identified in all consultations. The third most frequently coded BCT was 9.3 (comparative imagining of future outcomes), which was coded in 92% (*n* = 49) of all consultations. Other commonly coded BCTs include 4.2 (information about antecedents) (89%, *n* = 47) and 7.1 (prompts and cues) (70%, *n* = 37). Some less frequently coded BCTs included 1.3 (goal setting (outcome)), 1.9 (commitment), 5.4 (monitoring of emotional consequences), 6.2 (social comparison), and 12.2 (restructuring the social environment), which all only appeared in 2% (*n* = 1) of consultations (Fig. 1). Overall, there was no difference between the frequency and dosing of BCTs (see Appendix B).

### BCTs by trial consenters and decliners

**Fig. 2:**
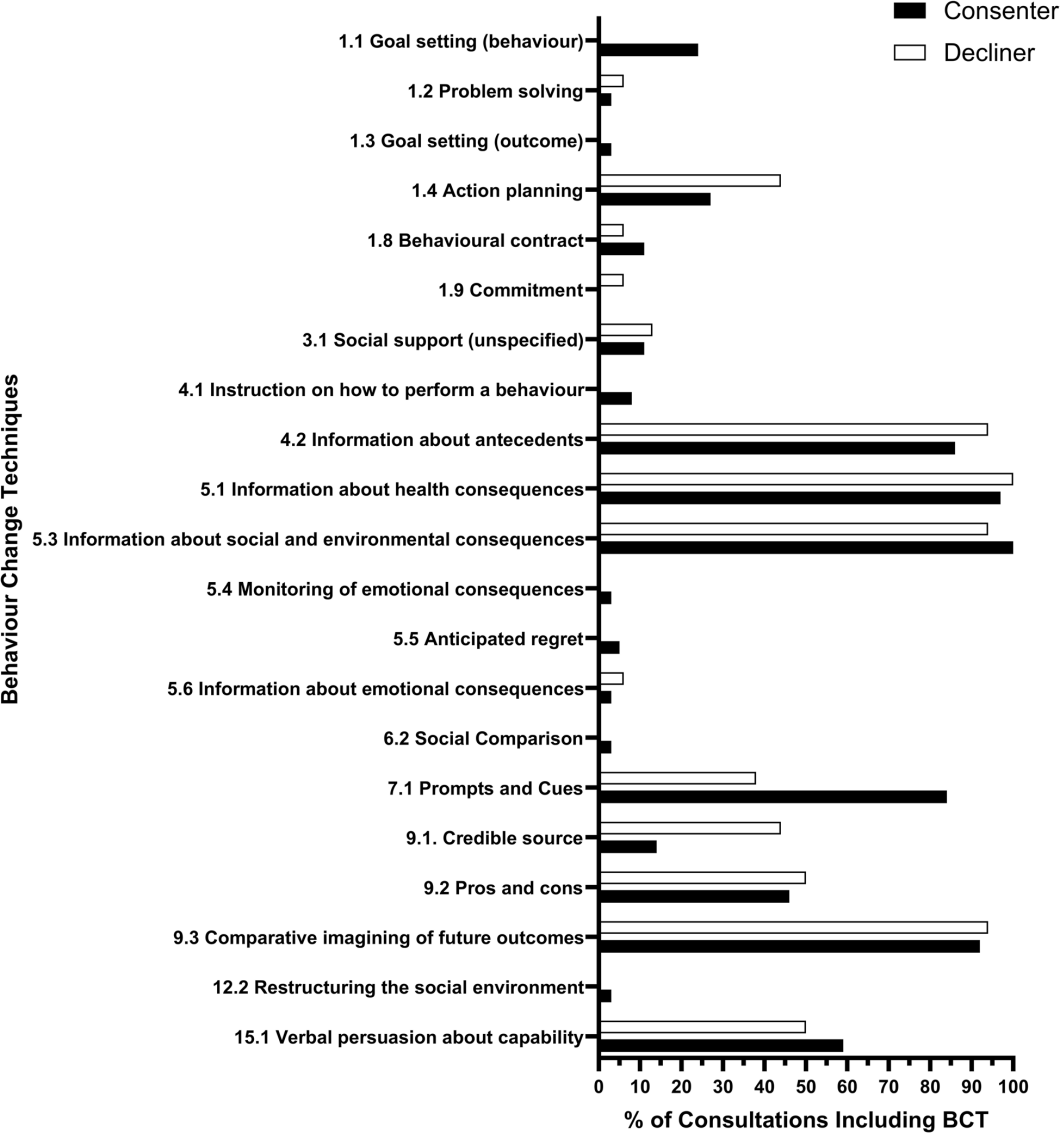
Frequency of BCT coding in consenters and decliner consultations

There was no apparent difference between the mean number of BCTs identified between trial consenters (*M* = 6.8, *SD*: 1.5) and decliners (*M* = 6.4, *SD*: 2.1). The frequency of BCTs was compared between consenters and decliners; BCT 7.1 (prompts and cues) was coded more frequently in consenters (84%) than decliners (38%). The BCTs 1.1 (goal setting (behaviour)), 1.3 (goal setting (outcome)), 4.1 (instruction on how to perform a behaviour), 5.4 (monitoring of emotional consequences), 5.5 (anticipated regret), 6.2 (social comparison), and 12.2 (restructuring the social environment) were only coded in a few trial consenter consultations. BCT 1.4 (action planning) was, however, coded more frequently in decliner consultations (44%) than consenter consultations (27%). Credible source (9.1) was also coded more in decliners (44%) than consenters (14%) (Fig. 2).

### BCTs by ‘action’

Table 4 illustrates the frequency of BCT coding instances within each ‘action’ as per the AACTT framework. The AACTT action with the highest frequency of BCTs was ‘Providing information about what involvement in the trial entails and trial related activities’ with 15 of the 21 BCTs identified present and a total of 120 BCT instances. The action with the least BCTs was ‘Exploring preferences’ with 5 of the 21 BCTs identified present and a total of 12 BCT instances.

**Table 4:** BCT coding frequencies to AACTT specified behaviours

		**AACCTT behaviours**								**Total**
		Discussing the option of taking part in the trial or not	Describing the trial interventions or alternatives (e.g. standard care), which may include describing trial rationale	Discussing the trial and/or the interventions in a balanced way	Exploring preferences (e.g. if the patient expresses a preference for an intervention or standard care, the trial staff probes into this/asks questions)	Providing information about what involvement in the trial entails and trial-related activities (e.g. questionnaires, visits, how to take the intervention)	Seeking consent from patients (e.g. consider voluntariness (i.e. not obliged) and autonomy (i.e. up to you to make decision))	Discussing or enacting (potential) randomisation	Exploring and/or checking patients’ acceptance of allocation (before or after randomisation) and includes potential to cross-over or withdraw	
**Behaviour change techniques (BCTs)**	1.1 Goal setting (behaviour)					**3**	**4**		**1**	**8**
	1.2 Problem-solving					**1**	**2**			**3**
	1.3 Goal setting (outcome)					**1**				**1**
	1.4 Action planning	**4**	**4**			**8**	**10**	**1**	**1**	**28**
	1.8 Behavioural contract					**1**	**5**			**6**
	1.9 Commitment						**1**			**1**
	3.1 Social support (unspecified)					**3**	**3**			**6**
	4.1 Instruction on how to perform a behaviour		**1**				**3**		**1**	**5**
	4.2 Information about antecedents	**14**	**35**	**6**	**4**	**6**	**21**	**22**	**10**	**118**
	5.1 Information about health consequences	**6**	**17**	**46**		**28**	**1**	**13**	**4**	**115**
	5.3 Information about social and environmental consequences	**16**	**29**	**11**		**37**	**4**	**40**	**8**	**145**
	5.4 Monitoring of emotional consequences								**1**	**1**
	5.5 Anticipated regret	**1**							**1**	**2**
	5.6 Information about emotional consequences			**1**		**1**				**2**
	6.2 Social comparison				**1**					**1**
	7.1 Prompts and cues		**2**	**2**		**15**	**31**	**1**	**1**	**52**
	9.1. Credible source	**6**	**2**	**4**	**1**	**1**				**14**
	9.2 Pros and cons			**24**		**1**				**25**
	9.3 Comparative imagining of future outcomes	**20**	**21**	**22**	**4**	**13**	**1**	**4**	**18**	**103**
	12.2 Restructuring the social environment					**1**				**1**
	15.1 Verbal persuasion about capability	**1**			**2**		**21**		**12**	**36**
**Total**		**68**	**111**	**116**	**12**	**120**	**107**	**81**	**58**	

### Length of consultation and frequency of BCTs

**Fig. 3:**
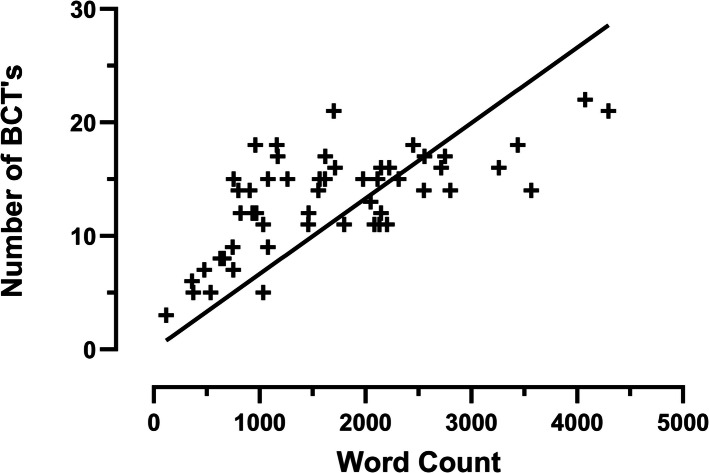
Number of BCTs plotted against word count

The number of BCTs coded and the word count of the coded content were significantly positively correlated, *r* (53) = 0.67, *p* < 0.001, and 95% *CI* (0.48, 0.79) (Fig. 3). Additionally, consultations that resulted in consent tended to be longer (*M*: 1748, *SD*: 940) than decliner consultations (*M*: 1519, *SD*: 990).

### The presence of SDM within consultations where trials are discussed

Across all three trials, total observer ratings using OPTION5 were rated as moderately low with a mean score of 27.7 (*SD*: 12.2) out of a possible 100 (Table 5). There was no difference between trials. On average, OPTION5 ratings were marginally higher in the sample of consenters (*M*: 29.0, *SD*: 13.2) than decliners (*M*: 25.0, *SD*: 9.3) across the three trials (Table 5).

**Table 5:** OPTION5 mean scores by trial

**Trial**		**Total rescaled mean score (SD)**						**Total rescaled mean score (SD)**	
A		26.3 (7.8)				Consenters		26.3 (7.6)	
						Decliners		-	
B		26.7 (12.1)				Consenters		32.5 (11.3)	
						Decliners		20.8 (10.7)	
C		30.0 (15.6)				Consenters		32.5 (21.0)	
						Decliners		27.5 (7.9)	

Overall, item 5 (integrating patient preferences) had the lowest average rating with a mean of 0 (*SD*: 0.2) in consenters and 0.1 (*SD*: 0.5) in decliners (Table 6), whereas item 1 (conveying the existence of options and need for a decision about trial participation) had the highest average ratings with a mean of 2.1 (*SD*: 0.9) in consenters and 1.9 (*SD*: 1.1) in decliners.

**Table 6:** OPTION5 scores by consenters and decliners

	**OPTION item** Mean (SD)					**Total rescaled mean score (0–100)**
	Item 1 (conveying existence of alternate options and need for a decision)	Item 2 (supporting deliberation about options)	Item 3 (providing information about risks and benefits of each option)	Item 4 (eliciting preferences)	Item 5 (integrating preferences)	
Consenters	2.1 (0.9)	0.8 (1.2)	1.8 (0.8)	1.1 (0.9)	0 (0.2)	29.0 (13.2)
Decliners	1.9 (1.1)	0.7 (1.1)	1.3 (0.5)	1.0 (1.0)	0.1 (0.5)	25.0 (9.3)

## Discussion

This study has demonstrated the feasibility of applying the BCT taxonomy and OPTION5 tool to consultations in which clinical trials are discussed. The application of the BCT Taxonomy identified a range of BCTs implicitly applied by healthcare professionals within clinical trial consultations with some variation of application between trial consenters and decliners. In addition, the OPTION5 tool identified low levels of SDM across trial consultations with little variance between trials or consenters and decliners.

The BCT coding identified 21 of 93 possible techniques that are being used by healthcare professionals in recruitment consultations across three clinical trials. Across all 53 included consultations, healthcare professionals provided potential participants with information about the health consequences (BCT 5.1) and social or environmental consequences (BCT 5.3) associated with taking part in the trial. Although there was no apparent difference in the average number of BCTs being implemented between consenters and decliners, healthcare professionals were more likely to use prompts and cues (BCT 7.1) in consultations resulting in a consent decision than a decline outcome, whereas credible source (BCT 9.1) was more likely to be used in consultations resulting in a decline decision. This is surprising as the existing literature [15] and the BCTTv1 suggest that the presence of a credible source would increase the likelihood of a consent decision. It is possible that healthcare professionals were citing credible sources as a final effort to unsuccessfully encourage uninterested patients to take part in the trial.

The most frequently occurring BCTs were those that involved information provision about the consequences of participation in the trial (BCTs 5.1 and 5.3). BCT 5.3, which involves the provision of information relating to social and environmental consequences, was particularly prevalent during discussions around randomisation. This is likely due to the requirement for trial staff to provide potential participants with all of the relevant information (e.g. possible adverse events or harm and what randomisation means in the context of a clinical trial) to make a fully informed decision, as outlined in the Declaration of Helsinki [16]. Similarly, participants are required to provide written, signed consent [8, 17]; in our coding, prompts and cues (BCT 7.1) frequently captured the provision of the informed consent form. It is possible that this BCT appeared more often in consultations that resulted in consent simply because those participants were already inclined to take part and so were presented with a consent form, whereas less interested participants would not have been provided the consent form. In such cases, the introduction of the consent form may have preceded, rather than influenced, the decision to participate, suggesting that its presence could be incidental rather than a driver of consent.

The OPTION5 ratings suggest that across all three included trials, SDM was low; this reflects minimal to moderate effort made by the healthcare professional to use SDM during recruitment consultations. Ratings of SDM were particularly low for item 5, which suggests that healthcare professionals made minimal to no effort to integrate patients’ preferences into decision-making. The AACTT specification identified that only 87% of the consultations across all three trials were coded under ‘discussing the option of taking part in the trial’. This could somewhat explain why SDM was rated as low, as it would be expected that consultations discussing clinical trial participation would include a discussion on the participants’ ability to choose whether to take part or not. Similarly, when comparing the BCT coding with the AACTT-specified behaviours, we can see that the action with the least coding instances was ‘exploring preferences’, suggesting healthcare professionals are employing fewer of these techniques to elicit and explore patients’ preferences relating to the trials. The action ‘providing information about what involvement entails, and trial related activities’ however had the highest number of coding instances. Though still a low score, item 1 (conveying existence of options and need for a decision) in the OPTION5 rating had the highest average scores, which suggests that healthcare professionals are encouraging more SDM when presenting options to the patient relating to the study. This alignment between the OPTION5 rating and the BCT-AACTT coding suggests that healthcare professionals are relatively frequently presenting options and information about the trial to their patients but are ‘worst’ at exploring the potential participants’ preferences relating to the study. Importantly, the existing literature suggests that exploring participant preferences can improve informed consent-related decision-making and recruitment to clinical trials [18, 19]. Similarly, the development of a new measure of participatory informed consent, the DEVPICv2, emphasises the importance of SDM principles, suggesting that engaging participants in discussions about their preferences is essential for achieving truly informed consent [20].

SDM was demonstrably low across all three trials. In a secondary analysis of interventions for increasing SDM [21], three BCTs were identified as associated with more effective SDM implementation when performed by healthcare professionals (2.2 feedback on behaviour, 4.1 instruction on how to perform a behaviour, 6.1 demonstration of the behaviour). Only 1 of these 3 BCTs (4.1) was identified in our analysis but only in 3 of the 53 consultations. This suggests that the BCTs potentially associated with effective SDM were not commonly identified in the three sampled trials. This is not all that surprising, considering the OPTION5 coding revealed that SDM was particularly poor across these trials.

### Implications

Given the potential role of SDM in ensuring informed choice when considering participation in a clinical trial and the critical role of SDM [8, 9], the findings of this study highlight the need for greater efforts to ensure that healthcare professionals consistently apply principles of SDM to support truly informed choices among potential research participants. For example, healthcare professionals could make additional efforts to elicit and integrate patient preferences when considering them as potential participants in a study. One such way that SDM could be improved is through the provision of decision aids and communication skills training to healthcare professionals. Decision aids have been shown to positively influence informed consent decision-making in clinical trials and are generally perceived as useful by trial staff and patients [22]. Previous literature also suggests that training healthcare professionals in communication skills can positively influence patient informed care and SDM [23].

Previously, the BCT taxonomy and measures of SDM have been implemented to better understand clinical trial recruitment by conducting interviews with trial recruiters [10, 24]. However, much of this literature [10, 24] relies on primarily qualitative or retrospective accounts from interest holders, typically healthcare professionals, rather than real-time observations. This study demonstrates the feasibility of capturing these interactions as they occur, offering more accurate and detailed insights into the consultation process. It also demonstrates how these two approaches to understanding the data can complement each other, with the potential to improve consultations in which trials are discussed.

### Strengths and limitations

One limitation of the present study concerns the length of consultations. In reviewing the AACTT-specified sections of each transcript, we observed that longer transcripts tended to include more behaviour change techniques (BCTs). This may reflect the fact that longer consultations offer more opportunities for BCTs to be present, rather than indicating a greater density of BCTs. Additionally, the sample contained more consultations from consenters than decliners, and consenter consultations tended to be slightly longer. This could also suggest that there were more opportunities for BCTs to be present in consenter consultations than in decliners.

This study was not powered to determine whether the implicit use of SDM or BCTs influenced participants’ decisions regarding trial enrolment. While our findings suggest they may play a role, future research should be designed to rigorously assess whether SDM and BCTs impact participation decisions.

It is important to note that SDM was not the primary aim of these recruitment conversations, and as such, elements of SDM may not be expected to feature prominently. Findings may vary across clinical areas, trial phases, and populations. Those facing higher stake medical decisions (e.g. altering cancer treatment plans) may need extra time to consider their treatment and trial participation, suggesting they could benefit most from SDM. Future research could usefully explore how the use of BCTs and SDM elements differs by gender and ethnicity, particularly in light of persistent inequities in trial participation despite targeted interventions. Similarly, our use of OPTION5 (which is coded retrospectively by observers as opposed to considering the experience of the healthcare professional or the potential participant) may not account for some non-verbal cues such as hesitation or emphasising certain words which may contribute to perceptions of SDM. Therefore, future research may wish to explore SDM using more direct methods.

Despite this, the present study used rigorous methods for the AACTT specification, BCT coding and OPTION rating. Each individual exercise was executed by at least two authors and checked by a third. Additionally, a key strength of this study was the use of audio-recorded consultations rather than relying on retrospective self-reports. This allowed for the capture of more natural, everyday interactions between participants and recruiters. As a form of secondary analysis, the data were collected independently of the current research aims, reducing the likelihood of observer effects or altered behaviour by recruiters. Moreover, secondary analysis facilitates collaboration across research teams, enabling broader insights from existing data.

## Conclusion

This study has demonstrated the feasibility of applying the BCT Taxonomy and OPTION5 framework to consultations in which clinical trial participation is discussed. The application of the BCT Taxonomy identified a range of BCTs implicitly used by healthcare professionals within clinical trial consultations, with some variation in their use between consenters and decliners. In addition, the OPTION5 tool identified consistently low levels of SDM across consultations, with little variation by trial or participant decision.

Together, these findings suggest that while behaviour change techniques are present and could improve recruitment discussion outcomes, their application is not yet intentional. Furthermore, the low SDM scores indicate limited support for potential participants in making informed, preference-sensitive decisions, despite the ethical requirements of trial participation decisions. These results point to opportunities for improving recruitment to clinical trials by integrating evidence-based behaviour change strategies alongside encouraging and practicing SDM. Future work could explore introducing interventions and decision aids that enable healthcare professionals to more effectively apply BCTs and SDM techniques. These improvements to recruitment practices will ultimately enhance both recruitment outcomes and the quality of participants’ decision-making in clinical trials.

## Supplementary Information

Additional file 1. Appendix A: Examples of Behaviour Change Technique.

Additional file 2. Appendix B: Dosing of BCTs.

## Data Availability

The datasets generated and/or analysed during the current study are not publicly available due to the nature of the data.

## Abbreviations

BCT: Behaviour change technique
BCTTv1: Behaviour Change Technique Taxonomy, version 1
SDM: Shared decision-making
AACTT: Action, actor, context, target, and time
OPTION5: Observing Patient Involvement
