# Supplementary material for: Shared decision-making and behaviour change collide: an analysis of consultations discussing clinical trial recruitment
Source: Trials. 2025 Nov 24;26:539. doi: 10.1186/s13063-025-09260-4 (PMC12642203; doi:10.1186/s13063-025-09260-4)
Supplement: Supplementary file 1 — Additional file 1. Appendix A: Examples of Behaviour Change Technique. [file 13063_2025_9260_MOESM1_ESM.docx]

| **Behaviour Change Technique** | **Coding rule for the present study** | **Example (trial)**  Underline = where BCT was coded |
| --- | --- | --- |
| 1.1 Goal setting (behaviour) | Set or agree on achieving a trial-based behaviour with the patient such as providing consent or starting the treatment. Goal must be a part of the intervention/treatment in the trial. | “…we give you the information sheet and give you a minimum of 24 hours to have a read of it, have a think about it, read the nature of it, but in essence it’s as I’ve described…you then say to me, probably over the phone, “Yes,” or, “No”” (B) |
| 1.2 Problem solving | Analyse or prompt patient to analyse the factors that could affect their behaviour and trial participation, by generating or suggesting strategies such as barriers and facilitators, asking questions. Problem solving must have a solution to be coded. | “Yes, a second pair of ears is always helpful. You will have remembered stuff that you won’t and vice versa. What I would say also - I think this is important - you’ll probably, even on the drive home, be like, “Oh, I wish I’d asked him that.” Just jot anything down” (B) |
| 1.3 Goal setting (outcome) | Set or agree on achieving the positive outcome of a trial-based behaviour such as stating what performing behaviour (e.g., filling in questionnaires) will hopefully lead to (e.g., show that they’re improving) | “I've got a few questionnaires for you to fill out at the moment. They’re just baseline questionnaires of how you feel on a day to day basis. Hopefully, it’ll show that you start to improve.” (A) |
| 1.4 Action planning | Prompt planning of achieving trial-related behaviour by detailing either the context, frequency, duration and intensity.  E.g.,  - Discussions of when/where or how long potential participants had to make an informed consent decision  - Scheduling appointments for treatment and/or assessments during the trial  - Advising the patient to take time to read through the patient consent form | “…if you want to do it, great, if you don’t want to do it, it makes no difference, you will still get the…  but if you take four months to decide then you can’t go in because there’s research time windows that we need to meet.” (C)  “…and you just come in every two months for a couple of years and have that.” (B)  “On the other hand if the computer says observation, wait and watch, what you’ll do is every three months we’ll ask you to come. We will suggest some diet and exercise, low fat diet and exercise.” (A) |
| 1.8 Behavioural contract | Create a written specification of the  behaviour to be performed (e.g., completing a consent form), agreed on by  the person (i.e., participant), and witnessed by another (i.e., healthcare professional). Only code when completing the consent form and not just when consent form is being provided or discussed. | “I’m going to ask you to have a look through that form and, if you agree to take part in the study, to initial down the side of each point and then to print your name and date and sign.” (B) |
| 1.9 Commitment | Ask the potential participant to affirm statements indicating commitment to take part in the trial or trial related behaviour | “…if you’re happy to take part in the study, you’ve had all your questions answered, you go, “Yes, I’d like to do so.”” (B) |
| 3.1 Social support (unspecified) | Advise the person to call a ‘buddy’, friend, relative etc. to encourage or support the trial-related behaviour  E.g.,  - Advising patient to ask friends/family to look through PIL’s and consent forms  - Advising patient to bring friends/family to appointments | “Probably best just to have some time, discuss it between yourselves and any extra family that you have, talk to them, and have a little bit of time to digest things because… talk to family, friends, look on the internet, whatever you needed to do.” (C)  “Talk to family, to friends, talk to, you can phone us up, talk to us again if you want to.” (C) |
| 4.1 Instruction on how to perform a behaviour | Advise or agree on how to perform a trial-related behaviour, such as how to complete a consent form or to read a participant information sheet | “So, what I need you to do is to initial in the boxes. You can see the boxes, can’t you?” (A)  “So, I guess the point today is it would be good if you could take that home and have a look at it in more detail. And then, what I’ll do is I’ll give you a call if that’s okay.” (B)  "Thank you. So obviously before you go away and read about, er, this, so this is the one I want you to take home and read about the study” (C) |
| 4.2 Information about antecedents | Provide information about antecedents (social and environmental situations, events, emotions and cognitions) that predict outcome/performance of behaviour.  E.g.,  - Provide information about the trial intervention and clarify that the trial is being conducted as they do not know the best thing to do currently  - Telling patient that they are under no obligation to participate  - Discussing with patient what leads to consent,  - Referring (but not providing) to consent forms, e.g., ‘I would then give you a consent form and ask you to sign it as a necessary for trial participation | “…there is national and international uncertainty as to which is the best, and that’s why we’re doing this trial, because clinical trials often answer those sorts of questions, because they give excellent care and we get excellent follow up so that we can then look at the results and we will then know in the future what is the best way to manage grade two meningioma. (C) – for trial  “But up until now we’ve never known what the right thing is to do; do you leave them alone and do they not cause any trouble, or should we be taking them all out and that’s what the trial is designed to do is to try an answer that question for us. (A) – for trial  “Um, so I think if you were interested in, in exploring, having radiotherapy at all, then I think you should have a think about the trial and whether you want to go into it.” (C) – for participation |
| 5.1 Information about health consequences | Provide information (e.g., written, verbal, visual) about the health consequences of performing the trial related behaviour (e.g., participating in study, being randomised into a condition, completing treatment)  E.g.,  - Health consequences and side effects of a specific treatment such as radiotherapy  - Health consequences associated with untreated illness/disease  - Health consequences of specific condition randomisation compared to another | “Secondly, the maintenance can have its own problems in terms of respiratory/sinus infections, etc., particularly after, actually, bendamustine, which is very effective. It, when you get to the end of the treatment and onto maintenance, does supress your immunity a fair bit.” (B)  “It can make you tired. It can make you feel a bit headache-y and some people can be a little bit queasy, although usually not too bad.” (C)  “Where they've already given you a, an interesting hairdo, you would lose a bit more hair in the areas that we treat.” (C) |
| 5.3 Information about social and environmental consequences | Provide information (e.g., written, verbal, visual) about the social and environmental consequences of performing the behaviour (e.g., randomisation, consenting into study or taking the treatment).  E.g.,  - Discussing the practicality and logistics of the procedures, discussing benefits of trial on future treatment options, discuss impact on science. | “And we’re hoping that some time in the future once we’ve got a lot of you know, thousands of people enrolled we’ll be able to say in the future, “Right, this is definitely what we need to do because it’s been proven by scientific data”. (A) – for trial  “As far as going back to work is concerned, I would certainly have thought that there would be no reason, if we put the whole radiotherapy thing to one side…… sort of timescale that you’re looking at anyway. I don’t see that there’s, that there would be any particular problem with that......Well, it, it kind of depends. The radiotherapy itself doesn’t take very long to do.” (C) – for participation |
| 5.4 Monitoring of emotional consequences | Prompt assessment of feelings after attempts at performing the trial related behaviour, such as asking patient how they feel about the condition they’re randomised to | “How do you feel about that? Bit disappointed. Would you rather have had the surgery?” (A) |
| 5.5 Anticipated regret | Induce or raise awareness of potential future regret about not performing trial related behaviour or participating in trial | “You know, say you said I don't want to go in the study and then you went away and it comes, you know, we did a scan and suddenly there's a tumour regrowing, you might say oh gosh... I wish I'd gone into it or you, you know...” (C) |
| 5.6 Information about emotional consequences | Provide information (e.g. written, verbal, visual) about emotional consequences of performing the trial related behaviour | “Now, there will be days where you’ll be like, “No, thanks, I don’t really fancy going for a walk today,” or whatever, but there will be days where, actually, it’s important to get out and about and… You know, actually, staying active is important because it will help your sleep, it will help your mood, it will help your appetite, it’ll help all the other things that are important in terms of feeling as normal as you can do when you’re going through something, you know, like this, really.” (B)  “But there is the anxiety, will it come back and when will it come back.” (C) |
| 6.2 Social Comparison | Draw attention to other people’s (or healthcare professional’s own) feelings or thoughts about the trial related behaviour to allow comparison with patients own behaviour or engagement with that behaviour | “Had the trial have been going when I had mine, I would have said, “No, I want the surgery” as you have done. But then I’ve put a little bit of weight on and now I’ve started to get the pain back. Obviously, there is no gall bladder there anymore. So, I’ve had a scan again.” (A) |
| 7.1 Prompts and Cues | Induce or provide information (giving the patient the Participant Information Leaflet or consent form) to cue or prompt trial related behaviour, | “It pains me to say. But what we like to do is to give you an information sheet to take away.” (C)  “So, all this applies and I’ll give you the leaflets. You can go away with those.” (A) |
| 9.1. Credible source | Present verbal or visual communication from or about a credible source in favour of the trial and associated trial behaviours, such as who is funding the trial or what the healthcare professional thinks of the trial | “So I think it is a very reasonable trial for you. I think it is a very justified trial in what they are trying to work out” (B)  “…in fact my colleagues in (Hometown), and ourselves, we often come together to discuss management in this situation. And I think most of us now feel that outside of the trial, we wouldn’t routinely offer radiotherapy” (C)  “Now I am presenting to you about a clinical trial where the government has funded us” (A) |
| 9.2 Pros and cons | Advise or help person to identify and compare reasons for wanting (pros) and not wanting to (cons) change or engage with the trial related behaviour | “Surgery comes with risks and benefits. The benefit is you go back to normal life, normal diet, pain free. It isn’t always the case. Some people do continue in pain, and we may have to investigate you for other things. You will be left with four little scars, but the scars will fade.” (A)  “They both have their pros and cons. You know, if we don't do anything, obviously you don't do anything and you just carry on with your scans. But there is the anxiety, will it come back and when will it come back” (C)  “The benefits of that is that if you take a large population of people, it keeps people in remission for longer, if you take a large population of people. But it does cause more infections and sometimes people have to stop it early because of infections and things” (B) |
| 9.3 Comparative imagining of future outcomes | Prompt or advise imagining and comparing of future outcomes depending on, for example, taking part in the trial or not and being in one condition/intervention group versus another | “So I hope that we offer that little bit extra and that you feel that you’re getting that little bit extra by taking part in the study. As I said, if you don’t take part, if you choose not to it does not affect your care” (B)  “Whatever you decide, we'll still be monitoring you with scans” (C)  “…then there are two pathways - an operative pathway, or a conservative pathway...now, the operative route, is obviously an operation…the conservative route, this is why we are doing the trial, this national trial.” (A) |
| 12.2 Restructuring the social environment | Change or advise to change social environment in order to perform or engage with trial related behaviour | “…but what I would say is I’d bring somebody first time because you won’t quite know how you feel.” (C) |
| 15.1 Verbal persuasion about capability | Tell the person that they can successfully perform and choose to perform the trial related behaviour, by empowering them to make the choices themselves | “But it is entirely your choice. You wouldn’t be pressurised into anything.” (B)  “If you didn’t want to go into the trial, and as I say, it is completely…… you know, it is up to you and a lot of people, things like the logistics of the radiotherapy…would be a, a very valid reason to say no. (C)  “Again, you are free to change your mind at any time.” (A) |
